# Proposition of an Energy Intake Estimating Scale through Item Response Theory

**DOI:** 10.3390/nu15214511

**Published:** 2023-10-24

**Authors:** Victor Nogueira da Cruz Silveira, Ana Karina Teixeira da Cunha França, Cleber Lopes Campelo, Patrícia Maria Abreu Machado, Alcione Miranda dos Santos

**Affiliations:** 1 Postgraduate Programme in Collective Health, Federal University of Maranhão, São Luís 65020-070, Brazil; ana.franca@ufma.br (A.K.T.d.C.F.); alcione.miranda@ufma.br (A.M.d.S.); 2 Higher School of Health Sciences—Bachelor of Nursing, State University of Amazonas, Nurse at Brazilian Hospital Services Company (EBSERH), Manaus 69850-000, Brazil; ccampelo@uea.edu.br; 3 Directorate of Technologies in Education, Federal University of Maranhão, São Luís 65080-805, Brazil; patricia.machado@ufma.br

**Keywords:** psychometrics, adolescent, eating, surveys, questionnaires

## Abstract

Background: Traditional methods for assessing individual energy consumption often involve lengthy and intricate procedures. This study aims to introduce an Energy Consumption Estimation Scale, utilizing Item Response Theory (IRT) for adolescents aged 18–19 years. Methods: This psychometric investigation applies IRT to 93 items extracted from a validated food frequency questionnaire. The study encompasses a representative sample of 2515 adolescents from the São Luís birth cohort in Brazil. The latent trait, energy intake, is derived using IRT and subsequently validated through hierarchical multiple linear regression modeling. Significance was established at *p* < 0.05. Results: A Samejima’s model was successfully fitted (CFI and TLI > 0.9 and RMSEA < 0.08), effectively capturing variations across all energy consumption levels. Factors associated with the latent trait demonstrate consistent behavioral patterns. Adolescents with higher energy intake exhibited increased consumption of dairy products, artificially sweetened beverages, and seasonal fruits and vegetables. Conclusions: The proposed Energy Consumption Estimation Scale demonstrates a reliable measurement of energy intake and serves as a practical and concise alternative for assessing energy consumption among adolescents. These findings suggest the potential for adapting similar models for different age groups and incorporating diverse food items based on the obtained results.

## 1. Introduction

Common food habits in adolescence are omission of meals and substitution for energy-dense and nutritionally poor snacks [1,2]. Studies about food consumption with children and adolescents verify the low consumption of fruits, vegetables, and greens, and high consumption of candies, fast foods, and sugar-sweetened beverages [3]. High consumption of these foods has been associated with negative outcomes in health and nutrition throughout life [3,4]. Thus, it is necessary to improve methods of dietary assessment. However, this is a complex analysis, and the available instruments present some hindrances in evaluation and/or quantification in food consumption [5,6]. 

The food frequency questionnaire (FFQ) and the 24 h-food recall (24hFR) are the most widely used methods of dietary assessment worldwide [7,8]. The FFQ is highly used due to its capability of estimating usual food consumption in longer periods of time and its ease of application [7]. However, FFQs can produce unreliable energetic and nutrient estimates due to their number of food items, limitation of the foods present in the FFQ list, or respondent’s memory [6,7,8]. Moreover, the FFQ assesses the respondent’s current food consumption, but this is an event with great variability [5,7,8,9]. 

Hence, because of these limitations, many areas have undergone advancements in measurement and psychometric methodologies have been applied to circumvent such problems [10,11,12]. To obtain suitable estimates, the Item Response Theory (IRT) has been suggested as a method to measure latent traits, an unobserved or not measurable characteristic of individuals or populations, in different areas of knowledge [12,13]. 

Moreover, unlike Classical Test Theory (CTT), the Item Response Theory (IRT) offers a more comprehensive and intricate insight into an individual item’s endorsement within a latent trait of interest [11]. This holds fundamental significance because food investigation approaches often rely on instruments with predefined food items. Additionally, IRT enables the positioning of both items and individuals along the latent trait, enhancing the reliability of assessing their food consumption [11].

Despite nutrition being an area with a vast number of latent traits, IRT is a recent resource in this area and can improve the accuracy of food consumption evaluation [14,15,16,17]. Therefore, this study aimed to develop a scale through IRT to estimate energy intake of adolescents from a north-eastern Brazilian city. 

## 2. Materials and Methods

This is a psychometric study that uses IRT analysis to develop a scale for estimating energy intake in Brazilian adolescents. Data of the participants of the third phase of the RPS cohort consortium were considerable. Methodological details of the RPS cohort consortium were published elsewhere [18]. 

The RPS birth cohort took place from March 1997 to February 1998 (when the children were born), baseline. The children were reassessed in 2005 (school age: between 7 and 9 years), and considered the first follow-up; and again in 2016, when they were in adolescence (18–19 years old), according to follow-up. To increase the power of the analysis and prevent future losses, the cohort was opened to include other individuals born in São Luís, Maranhão, making a final sample consisting of 2515 adolescents aged 18 to 19 years, evaluated in the year 2016 [18]. 

The study sample consists of 2515 participants of the RPS cohort. The participants responded to a questionnaire containing socioeconomic and demographic data, anthropometric measures, biochemical data, lifestyle habits, and food intake.

The socioeconomic and demographic variables for this study were: gender (male and female), educational level (currently studying and not studying), beneficiary of government program (yes and no), number of residents in the household, marital status of the respondent (single and compromised) and their parents (married and divorced), self-reported skin colour as a proxy of race/ethnicity (white and non-white), and per capita family income (<¼ of minimum wage and ≥¼ of minimum wage). 

In addition, anthropometric data as body mass index, which was obtained through weight in kilograms divided by height in square metres (m^2^) was used. Body weight, percentage of free fat mass, and percentage of body fat which were obtained through air displacement plethysmography device (BOD POD, COSMED USA Inc., Concord, CA, USA). Height was obtained through a portable stadiometer (Alturexata^®^, Belo Horizonte, Brazil) following techniques recommended by the World Health Organization (WHO) [19]. 

Biochemical data such as total cholesterol, high-density lipoprotein cholesterol (HDL-c), low-density lipoprotein cholesterol (LDL-c), very low-density lipoprotein cholesterol (VLDL-c), and triglycerides were also collected. For biochemical analysis, 40 mL blood samples were obtained from the cubital vein aseptically by an experienced technician in this procedure and were analysed by the automated enzymatic colorimetric method using Roche^®^ Cobas c501 equipment (Indianapolis, IN, USA). 

Dietary data were obtained through a semiquantitative food-frequency questionnaire concerning the usual food consumption in the last twelve months. This FFQ was adapted from an instrument validated previously [20]. For this study, the portion sizes were changed and excluded/included regional food items which are common to the local adolescent population.

The adapted FFQ included ninety-three food items arranged in seven food groups, the options of consumption frequency during the last year and the size of the mean reference portion, so that the present study could estimate whether the portion usually consumed was small, equal, or larger than the reference. The average time to apply the FFQ was approximately 40 min. The methodology of the adapted FFQ relative validity and application in the adolescent population was previously described by Bogea et al [21]. It also presents lifestyle variables, such as physical activity practice, the daily habit of replacing meals with snacks, skipping meals, going to restaurants daily, use of supplements, and being on a diet. 

Initially, the ninety-three food items were split into nine food groups according to their nutritional similarities, then three ordered response categories for each food group were assigned based on their daily referred consumption of every food item. Category 0 (lower) represents the non- or less than a portion consumption of the food item, category 1 (intermediate) represents the consumption of one portion and category 2 (higher), the consumption of more than one portion. 

For the item’s generation, the sum of responses for each group were calculated, then calculated the mean according to the number of the food items in the group, making up the mean score for each group (Table 1). The latent trait was defined as the energy intake of Brazilian adolescents. 

For the study, latent trait estimates were considered unidimensional IRT models. The dimensionality of the adapted FFQ was carried out on the polychoric correlation matrix and principal components with a parallel analysis through the psych package [22]. The polychoric correlations are suitable coefficients for items with ordinal scales [23]. In addition, dimensionality was also studied using the confirmatory factor analysis.

Different models for ordered response categories were adjusted to estimate the item’s parameters. For the adjustment of the model, the following indexes were analysed: comparative fit index (CFI), Tucker–Lewis index (TLI), and root mean square error of approximation (RMSEA). As adjustment criteria, the following values were adopted: CFI greater than 0.90, TLI greater than 0.90, and RMSEA from 0.05 to 0.08 [24]. The model with a lower Akaike information criterion (AIC) was selected.

The graded response model (GRM) presented the lower AIC. So, from this model, the discrimination parameter (*a_i_*) and location parameter of the *k*-th category of item *i* (*b_i,k_*) for each item were obtained. The GRM is represented by the following equation [25]:

$$P_{i,k}^{+}=\frac{1}{1+e^{-a_{i}(\theta_{j}-b_{i,k})}}$$

where *i* denotes the number of items in the instrument (*i* = 1, …, 9); j is the total number of respondents; *k* is the number of categories of the items minus 1; *θ_j_* is the latent trait (energy intake) of the *j*-th respondent, and $P_{i,k}^{+}$ is the probability of the *j*-th respondent with a given energy intake of *θ_j_* to be ranked in a certain category of the *i*-th energy intake level or higher. It is desirable that the items have *a_i_* > 0.7 and −3 < *b_i,k_* < 3. Items with values of *a_i_* ≥ 1 are considered with a reasonable power of discrimination [26].

The construction of the scale of energy intake was performed by positioning the response categories of each item at a cumulative probability point of 0.50 (i.e., probability that the adolescents would consume a food item according to a specific item category or superior).

Linear transformation of the latent trait was used to avoid negative numbers and to facilitate the discussion of the results [27]. Usually, the latent trait is set on the scale (0, 1), therefore with a mean of zero and standard deviation of one. In this study, the latent trait was transformed into scale (400, 100); that is, with an average of 400 and standard deviation of 100.

To provide an example of a potential use of the estimate of energy intake through IRT, a hierarchical linear regression model to evaluate the association between the latent trait (dependent variable) and related factors with the energy intake was adjusted. These factors were selected due to their relationships with energy intake. Therefore, the factors were grouped in three blocks: 

Block 1: educational level, number of residents in household, gender, marital status, skin colour, parent’s marital status, family income, and participation in an income transfer government program;

Block 2: body mass index, percentage of body fat, total cholesterol, HDL-c, LDL-c, VLDL-c, and triglycerides;

Block 3: daily breakfast consumption, daily lunch consumption, daily trip to restaurant, exchange lunch for snack, exchange breakfast for snack, use weight gain supplement, use weight loss supplement, diet for weight gain, diet for weight loss, exercise practice for weight gain, and exercise practice for weight loss. 

The explanatory variables were inserted according to their hierarchical level. All variables adjusted to each other at each level, and those that presented with a significance lower than the 20% level were maintained for the next block. Statistical significance was set at 5% in the final model. 

Numerical variables were described in means and standard deviation or medians and interquartile range and categorical in counts and percentages. All the analyses were performed in R Studio [28]. The current study used the GIFI and PSYCH package for principal component analyses and MIRT package for IRT analyses.

The study was conducted according to the directives established in the Declaration of Helsinki, and all procedures involving human beings were approved by the Research Ethics Committee of the Federal University of Maranhão, Brazil under protocol number 1.302.489. All respondents gave their consent prior to the inclusion in the study.

## 3. Results

The sample was mostly made up of girls (52.45%) with a median BMI of 21.2 kg/m^2^. Most of the sample was not on a diet at the time of the survey (66.75%), but of those who were, had weight loss as their main objective (9.15%) (data not presented in table).

The principal component analyses showed a first factor with an accounted variance of 33%, supporting the unidimensionality assumption, i.e., showed a dominant dimension. Furthermore, the GRM model presented CFI of 0.96, TLI of 0.93, and RMSEA of 0.06 (IC95% 0.06; 0.07). The sociodemographic information, biochemical exams, and lifestyle habits of the individuals can be seen in Table 2.

The nine items from the adapted FFQ presented satisfactory discrimination power, with “meat and eggs” as the best indicator for differentiating the energy intakes of adolescents along the continuum (*a_i_* = 2.25). On the other hand, alcoholic beverages presented the lowest ai. Discrimination and location parameters with their respective standard errors (SE) are displayed in Table 3. 

Concerning the location parameters, the item “cereals and tubers” had the lowest value in the intermediate response category (*b_1_* = −1.81), implying that it is not necessary to have a high energy intake to consume a portion or more of the foods that compose this item. The item “candies” had the lowest location parameter in the higher category (*b_2_* = 2.14), indicating that the adolescents with high energy intakes easily consumed more than a portion of this food group in comparison with the others. Among the highest location parameters in both categories, it was observed that alcoholic beverages led in the intermediate category (*b_1_* = 2.09) and the dairy products in the highest (*b_2_* = 4.43), demonstrating that adolescents needed a high energy intake to consume a portion or more of the first and more than a portion of the second, respectively. 

In the final regression model, six factors were associated with the estimate of energy intake (latent trait). BMI was negatively associated with the latent trait (β = −1.544; *p* = 0.005) and replacing breakfast (β = 12.101; *p* = 0.003) and lunch (β = 19.074; *p* < 0.001) with snacks were positively associated (Table 4). The associated factors with the latent trait confirm the behaviour of energy intake within different situations. 

The GRM obtained comprises the whole latent trait, but it is predominantly appropriate for assessing energy intakes ranging from −3.2 to 5.9. Nevertheless, the model indicates that scores between −0.75 and 2.81, with a slight reduction in score 1.0, are more explicative than those in high and, especially, low levels, because the higher the score of information and the lower the SE, the greater the accuracy of energy intake estimates.

In Table 5 are presented three levels of scale (400, 100): low (0 ≤ *θ* ≤ 200); moderate (300 ≤ *θ* ≤ 500) and high (*θ* ≥ 600). Increases in the levels indicate higher energy intake. The first level (0 ≤ *θ* ≤ 200) demonstrates a low energy intake. The highest level (*θ* ≥ 600) demonstrates a diet rich in energy. Through the sequence observed in the item’s positioning, it is possible to identify the most consumed items in each level according to its response category.

Considering the response category 1 (consuming one portion), it was observed that “dairy products”, “fruits, vegetables and greens”, “seasonal fruits, vegetables and greens”, “meats and eggs”, and “sugar-sweetened beverages” were positioned in the moderate level (*θ* = 300), although with the consumption of one portion. The item “alcoholic beverages” was either positioned in the moderate level, but in *θ* = 500. On the other hand, the item “cereals and tubers” was the only one positioned in the low level (*θ* = 200). When observing the prominent level (*θ* = 800), the “dairy products”, “seasonal fruits, vegetables, and greens”, and “sugar-sweetened beverages” were the ones in the highest energy intake level.

## 4. Discussion

This study brings the novelty of developing a scale to estimate energy intake through a GRM of IRT analyses. Principal components analyses indicated that one factor explained most of the variance of the items. Previous studies addressed the vulnerabilities of FFQ, especially its dimensionality [5]. Using some food lists from FFQ to evaluate food consumption could be multidimensional because of the different nutrients and energy estimates assessed [29].

In the analysis of the FFQ, this study chose to group the items according to their nutritional similarities to exclusively assess their energy contribution to the subjects. By reducing the food list from ninety-three to nine items, it was possible to determine if the items would load on a single dimension. Prior studies applied IRT in diet quality [17,30], healthy eating [31], eating behaviour [14], and health motivation food choices [32], but only one used the FFQ as the study instrument, however investigating a different latent trait [30]. 

The test information curve showed that the model comprises the entire energy intake scale, but is more accurate in medium scores, with a slight reduction in score 1.0. The discrimination parameters obtained were adequate (*a_i_* > 0.65), although the item ‘Alcoholic beverages’ presented discrimination power below the cut-off (*a_i_* = 0.63). However, it was not excluded, because alcoholic beverages do not contribute significantly to energy consumption, but individuals with tendencies to consume it usually nibble on energy-dense foods while drinking [33], which justifies its permanence in the model.

Regarding the location parameters, in the intermediate category, the item ‘Cereals and tubers’ presented the lowest value, demonstrating that the adolescents did not need to have a high energy intake to eat one portion of this food group. This may be explained by Brazil’s dietary patterns, which are rich in rice during two meals a day, and other food items that comprise this group for breakfast [33,34,35,36]. The highest value in the intermediate category belonged to the alcoholic beverages, which is consistent with the low caloric contribution from the food items in this group [37,38], therefore requiring higher energy intake to endorse its consumption. In the higher category, the item ‘Candies’ presented the lowest location parameter, which means that the adolescents with lower energy intake values had a higher probability of consuming more than one portion of this group. Probably, this can happen because of the small size of this group portion [21], therefore making it easily consumable in greater quantities, or the propensity to consume more during adolescence.

In this study, a linear regression to evaluate the consistency of the latent trait was performed; that is, if the latent trait is measuring energy intake properly. In the final linear model, BMI was the only variable inversely associated with the latent trait. This probably occurs due to nutritional education and/or change of food habits, since the sample showed an opposite association in the percentage of fat free mass [39]. Proper eating habits can assist with healthy food choices which contribute to energy and macro- and micronutrients, thus reducing body weight or increasing free fat mass [3,4,30,32]. There is also the possibility that this behaviour of the BMI derives from reverse causality [40,41].

The per capita family income equal or superior to 25% of the current minimum wage in 2015 (R$788.00 [Brazilian currency] or US$299.17 [US currency]) showed a positive association with the latent trait, meaning that individuals with higher incomes consumed more energy than those below the cut-off. This is consonant with previous findings in Brazil [42,43,44], Japan, and high-income countries [45] where household income directly interferes with the amount of food, and therefore the energy consumed. Furthermore, besides the family income increasing energy intake, it is also capable of differentiating the source of calories, with refined carbohydrates the main source described for low-income families and proteins for medium- and high-income households [44,45].

Finally, being on a diet for weight gain and bad eating habits as replacing breakfast and lunch for snacks were positively associated with the latent trait. The former is compatible with the expected positive energy balance for weight gain, thus being the main finding to assess the latent trait [4,46,47]. The latter also explains the increase in energy intake (latent trait) [33,34,35,36,44,47,48]. A Brazilian’s breakfast may comprise coffee, bread, cheese, butter, and fruits, while the lunch is composed of rice, beans, some protein, and salad [34,35,36], and replacing these foods that are usually in natura for savoury snacks, sweets, sugary drinks, and/or ultra-processed foods in general not only contribute to higher energy intake but can also lead to unfavourable health outcomes [47,48]. 

One capability of IRT models is to estimate the probability that a specific respondent can provide a response category of an item and distribute it along the latent trait [5,14,27,30]. So, placing the items in a continuum according to their cumulative probabilities makes it possible to identify food consumption habits that result in a higher energy intake. In the current study, adolescents with low energy intake are more likely to consume one portion of cereals and tubers than those in moderate and elevated levels. 

In the response category 1 (consume one portion), most items were placed in the moderated intake level, but it was noticed that the items ‘Candies’ and ‘Various Foods’ were slightly above those items considered healthier (except for ‘Sugar-Sweetened Beverages’), meaning that, due to its consumption by the respondents, more energy intake is needed, consistent with their nutritional profile being energetically dense and nutritionally poor [49,50,51]. Additionally, the item ‘Alcoholic beverages’ was the most positioned on the right, thus requiring more energy intake for consumption. A systematic review with meta-analysis showed that the consumption of alcoholic beverages significantly increases both food energy intake and total energy intake, so in addition to its isolated consumption, food habits with high intake of alcoholic beverages can also be responsible for a greater energy intake [52]. 

In the response category 2 (consume more than one portion), all items were placed in the high energy intake level. Both ‘Fruits, Vegetables and Greens’ and ‘Cereals and Tubers’ items were placed in the lowest portion of this level, because Brazilian dietary patterns comprise the regular consumption of the foods that compose these food items within a day [33,34,35,36]. Regarding the items on the highest portion of energy intake, ‘Dairy products’ possibly were positioned there due to its high protein and fat content, as well as ‘Sugar-Sweetened Beverages’, which can be defined as a source of empty calories, rich in energy, with no micro- and macronutrients [53,54], thus strongly needing a high energy intake for its consumption. Regarding the item ‘Seasonal fruits, vegetables, and greens’, as contradictory as the need for greater energy intake may seem, a systematic review with meta-analysis has already shown that there is higher consumption of fruits and vegetables immediately post-harvest, and it may be a consequence of the great availability of these foods and/or low prices. Yet, it was also observed that seasonality is highly correlated with energy intake [55].

Previous investigations [56] reinforce the good assessment capacity of Item Response Theory on constructs involving food and nutrition. The reduction in the number of items makes the evaluation process faster and with a better capacity to evaluate latent traits, as well as allowing investigations into food and nutrition, especially in the primary healthcare process, to be easily carried out to prevent or mitigate effects of bad lifestyle habits.

This study has limitations, as the sample comprises only adolescents between 18 and 19 years, not allowing us to infer the results found here for all ages in adolescence. However, with a small age range, this study can minimise the variability of food intake inherent to this phase of life. On the other hand, the present study has an expressive and representative sample size, therefore minimising the occurrence of random error, reinforcing the reliability of the analysis, and increasing the power of the test. Furthermore, defining the items as the mean consumption portion of the food items present in the FFQ according to their nutritional similarity, reducing the number from ninety-three to nine, allowed us to exclusively quantify energy intake, as brief scales are better suited to evaluating latent constructs and not macro- and micronutrient estimates.

## 5. Conclusions

This study proposed an energy intake scale for adolescents aged between 18 and 19 years through an IRT model. From an adapted FFQ, the current study could arrange its food items in nine groups according to their nutritional similarities, thus avoiding evaluating nutritional estimates, and to evaluate a latent construct, namely energy intake. The present scale measured energy intake properly with better estimates in medium latent traits. The items were befittingly positioned on the scale, displaying in which latent trait level there was a ≥50% probability of consuming that food group according to each response category. Therefore, the energy intake scale can be applied to other populations, as the application instrument consists of mean portions of consumption of each food group present in FFQs. Thus, using an instrument with a smaller number of items and without the need for prior specification of nutritional estimates inherent to each food/food group consumed, a robust estimate of the energy consumed by the investigated population can be obtained, especially in scenarios of primary healthcare for individuals and populations, since the research process takes less time.

## Figures and Tables

**Table 1:** Description of each food group used as items in Item Response Theory analysis.

Item	FFQ Food Items
Cereals and tubers	Rice, whole grain bread, white bread, instant noodles, pasta, cassava flour, biscuits, cakes, potatoes, *tapioca*, beans
Dairy products	Milk, sugary milk, yoghourts, cheese, cream cheese
Fruits, vegetables, and greens	Orange, banana, papaya, apple, or pear, *açaí*, watermelon, pineapple, grapes
Seasonal fruits, vegetables, and greens	Avocado, mango, guava, lettuce, tomato, chayote, cabbage, West Indian gherkin, pumpkin, cucumber, pea pod, beet, onion
Meat and eggs	Beef, pork, poultry, fish, canned fish, sashimi, sushi, shrimp, crab, offal, hamburger, sausage, mortadella, bacon, eggs, butter, margarine, mayonnaise
Candies	Ice cream, candies, milk cream, fruit jams, chocolate bars, cocoa, stuffed cake
Sugar-Sweetened Beverages	Soda, artificial juices, fruit juices, soft drinks, coffee, sugary coffee, guarana of Amazônia
Alcoholic beverages	Energy drink, beer, wine, sugar cane rum
Various foods	Savoury, pizza, sandwich, kebab, popcorn, preserves, ketchup, breakfast cereals, cereal bars, nuts.

**Table 2:** Descriptive table of the adolescents (*n* = 2515).

Variables	*n* = 2515 ^1^
Currently studying	
No	767 (30.50%)
Yes	1748 (69.50%)
Number of residents in household	4.00 (3.00–5.00)
Gender	
Female	1319 (52.45%)
Male	1196 (47.55%)
Respondent marital status	
With partner	93 (3.70%)
Single	2422 (96.30%)
Skin colour	
White	495 (19.78%)
Non-white	2007 (80.22%)
Respondent’s parents’ marital status	
Married	1290 (51.29%)
Divorced	1225 (48.71%)
BMI	21.2 (19.1–24.0)
Percentage of body fat	21 (12–30)
Total Cholesterol	155 (135–176)
HDL-c	48 (41–56)
LDL-c	87 (71–105)
Triglycerides	79 (60–106)
Beneficiary of government program	
Yes	524 (20.83%)
No	1991 (79.17%)
Per capita family income	
<¼ of minimum wage	735 (29.25%)
≥¼ of minimum wage	1778 (70.75%)
Eat breakfast daily	
Yes	1942 (77.52%)
No	563 (22.48%)
Eat lunch daily	
Yes	2398 (95.77%)
No	106 (4.23%)
Go to restaurant daily	
Yes	17 (0.68%)
No	2488 (99.32%)
Replace breakfast with snack	
No	1762 (70.34%)
Yes	743 (29.66%)
Replace lunch with snack	
No	1797 (71.74%)
Yes	708 (28.26%)
Use supplement for weight gain	
No	2302 (91.53%)
Yes	213 (8.47%)
Use supplement for weight loss	
No	2483 (98.73%)
Yes	32 (1.27%)
Diet for weight gain	
No	2411 (95.86%)
Yes	104 (4.14%)
Diet for weight loss	
No	2285 (90.85%)
Yes	230 (9.15%)
Exercise for weight gain	
No	2281 (90.70%)
Yes	234 (9.30%)
Exercise for weight loss	
No	2191 (87.12%)
Yes	324 (12.88%)

^1^ Categorical variables are represented as *n* and %. Continuous variables are represented as medians and IQR.

**Table 3:** Mean portions and standard deviation (SD), discrimination (*a_i_*) and location (*b_1,2_*) parameters estimated by Item Response Theory analyses for adolescent energy intake estimation (*n* = 2515).

Item	Mean (SD)	*a_i_* (SE)	*b_1_* (SE)	*b_2_* (SE)
Cereals and tubers	0.94 (0.38)	1.68 (0.10)	−1.81 (0.08)	2.48 (0.11)
Dairy products	0.63 (0.50)	1.30 (0.08)	−0.50 (0.05)	4.43 (0.27)
Fruits, vegetables, and greens	0.78 (0.56)	1.23 (0.07)	−0.93 (0.06)	2.52 (0.12)
Seasonal fruits, vegetables, and greens	0.63 (0.52)	1.14 (0.07)	−0.51 (0.05)	4.13 (0.24)
Meat and eggs	0.78 (0.44)	2.25 (0.14)	−0.93 (0.04)	2.98 (0.13)
Candies	0.73 (0.57)	1.84 (0.10)	−0.60 (0.04)	2.14 (0.08)
Sugar-sweetened Beverages	0.74 (0.47)	1.12 (0.07)	−1.06 (0.07)	4.25 (0.26)
Alcoholic beverages	0.31 (0.61)	0.63 (0.06)	2.09 (0.20)	4.14 (0.39)
Various foods	0.67 (0.51)	2.01 (0.11)	−0.52 (0.04)	2.82 (0.12)

SE, standard error.

**Table 4:** Hierarchical linear regression model for consistency checking of the latent trait (*n* = 2515).

Variables	Block 1		Block 2		Block 3		Final Model	
	β	*p*	β	*p*	β	*p*	β	*p*
Currently studying	0.664	0.86	-	-	-	-	-	-
Number of residents in household	0.536	0.64	-	-	-	-	-	-
Male gender	10.609	0.003	−9.589	0.21	-	-	-	-
Respondent is single	4.183	0.65	-	-	-	-	-	-
Non-white skin colour	0.234	0.96	-	-	-	-	-	-
Respondent’s parents divorced	−2.271	0.52	-	-	-	-	-	-
Not beneficiary of government program	4.631	0.29	-	-	-	-	-	-
Per capita family income ≥¼ of minimum wage	13.327	0.001	13.375	0.001	12.139	0.003	12.560	0.002
BMI	-	-	−2.688	0.009	−1.577	0.006	−1.544	0.005
Percentage of body fat	-	-	−0.037	0.92	-	-	-	-
Percentage of free fat mass			1.366	0.003	0.838	<0.001	0.851	<0.001
Total cholesterol	-	-	−0.041	0.86	-	-	-	-
HDL-c	-	-	0.373	0.20	0.303	0.06	0.306	0.06
LDL-c	-	-	0.075	0.75	-	-	-	-
VLDL-c	-	-	0.574	0.36	-	-	-	-
Triglycerides	-	-	−0.047	0.73	-	-	-	-
Do not eat breakfast daily	-	-	-	-	−4.881	0.27	-	-
Do not eat lunch daily	-	-	-	-	−13.410	0.15	−14.850	0.10
Do not go to restaurant daily	-	-	-	-	21.797	0.29	-	-
Replace lunch with snack	-	-	-	-	18.948	<0.001	19.074	<0.001
Replace breakfast with snack	-	-	-	-	12.975	0.002	12.101	0.003
Use supplement for weight gain	-	-	-	-	−0.064	0.99	-	-
Use supplement for weight loss	-	-	-	-	−12.083	0.51	-	-
Diet for weight gain	-	-	-	-	25.017	0.008	24.333	0.009
Diet for weight loss	-	-	-	-	−11.993	0.08	−10.370	0.11
Exercise for weight gain	-	-	-	-	12.225	0.08	12.232	0.06
Exercise for weight loss	-	-	-	-	5.890	0.33	-	-

**Table 5:** Item positions in the energy intake scale developed from a graded response model through Item Response Theory analysis (metric 400, 100).

Response Category	Low			Moderate			High		
	0	100	200	300	400	500	600	700	800
1—One portion			CT	DP FVG SFVG ME SSB	C VF	AB			
2—More than one portion							FVG C	CT ME AB VF	DP SFVG SSB

CT, cereals, and tubers; DP, dairy products; FVG, fruits, vegetables, and greens; SFVG, seasonal fruits, vegetables, and greens; ME, meats and eggs; C, candies; SSB, sugar-sweetened beverages; AB, alcoholic beverages; VF, various foods.

## Data Availability

Data are unavailable due to privacy or ethical restrictions.
